# Erratum: YAP determines the cell fate of injured mouse hepatocytes *in vivo*

**DOI:** 10.1038/ncomms16146

**Published:** 2017-08-07

**Authors:** Norio Miyamura, Shoji Hata, Tohru Itoh, Minoru Tanaka, Miki Nishio, Michiko Itoh, Yoshihiro Ogawa, Shuji Terai, Isao Sakaida, Akira Suzuki, Atsushi Miyajima, Hiroshi Nishina

Nature Communications 8 Article number: 16017 ; DOI: 10.1038/ncomms16017 (2017); Published: 07 06 2017; Updated: 08 07 2017.

The financial support for this Article was not fully acknowledged. The Acknowledgements should have included the following:

This work was supported by a Nanken-Kyoten grant from Tokyo Medical and Dental University (TMDU).

