# Supplementary material for: YAP determines the cell fate of injured mouse hepatocytes in vivo
Source: Nat Commun. 2017 Jul 6;8:16017. doi: 10.1038/ncomms16017 (PMC5504293; doi:10.1038/ncomms16017)
Supplement: Supplementary Information [file ncomms16017-s1.pdf]

Type of file: PDF
Size of file: 0 KB
Title of file for HTML: Supplementary Information
Description: Supplementary figures and supplementary table.

Type of file: MP4
Size of file: 0 KB
Title of file for HTML: Supplementary Movie 1
Description: 3D movie showing engulfment of YAP (5SA)-expressing hepatocyte by Kupffer cells. 3D movie of the liver sections stained with anti-Myc (red) or anti-F4/80 (green) on day 3 post-HTVi (60x objective lens) for 20 seconds. 0-12 seconds: plus DAPI (blue); 12-20 seconds: minus DAPI.

Type of file: PDF
Size of file: 0 KB
Title of file for HTML: Peer review file
Description:

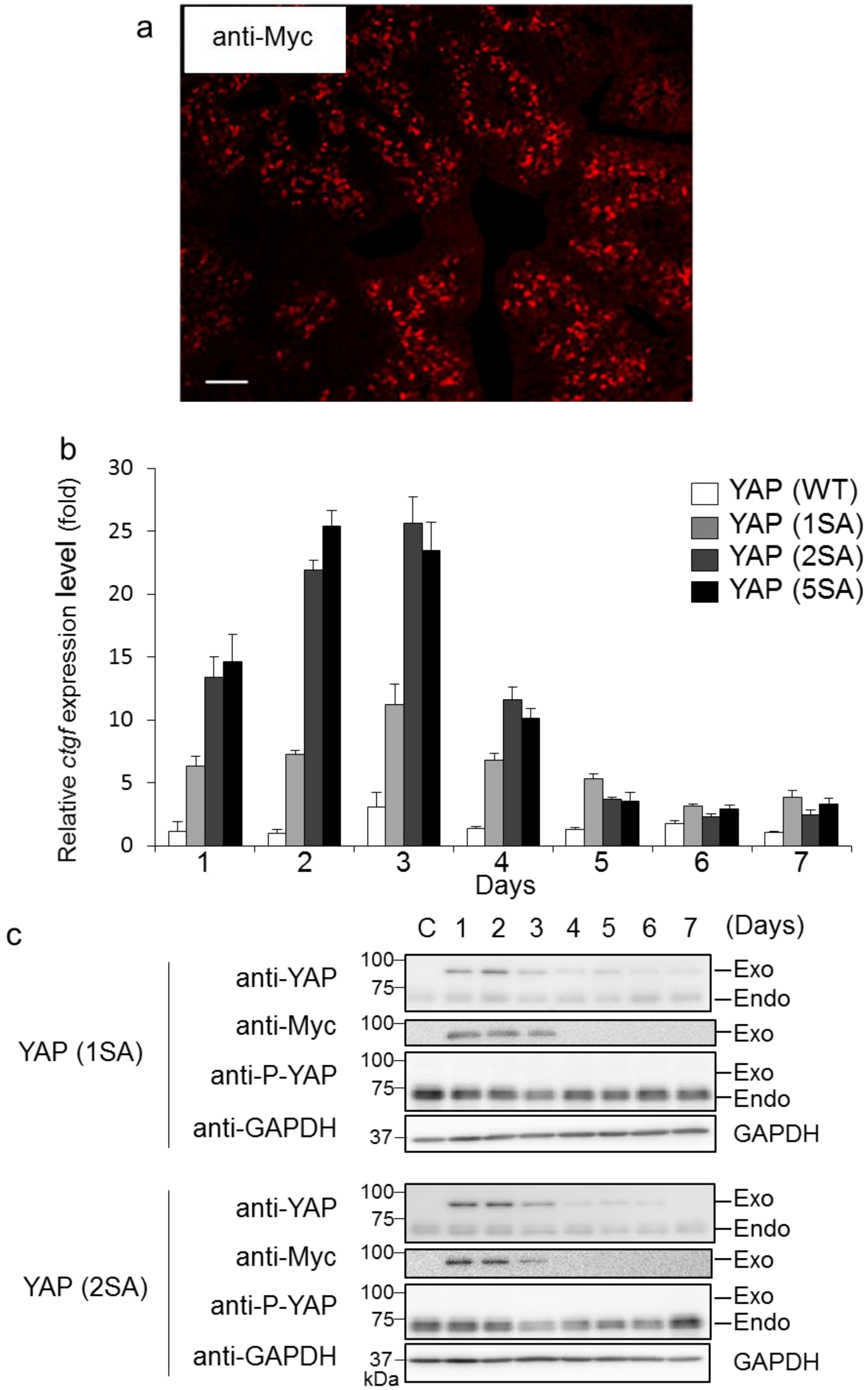
a
anti-Myc
b
Relative ctgf expression level (fold)
30
25
20
15
10
5
0
1
2
3
4
5
6
7
Days
YAP (WT)
YAP (1SA)
YAP (2SA)
YAP (5SA)
c
C 1 2 3 4 5 6 7 (Days)
YAP (1SA)
anti-YAP
anti-Myc
anti-P-YAP
anti-GAPDH
100
75
37
Exo
Endo
GAPDH
YAP (2SA)
anti-YAP
anti-Myc
anti-P-YAP
anti-GAPDH
kDa

Supplementary Figure 1. ***In vivo*** **mosaic analysis of YAP by HTVi in mouse liver.**

(a) Representative confocal immunofluorescence image of a liver section that was prepared from a mouse expressing Myc-tagged YAP and stained with anti-Myc antibody. Scale bar: 200 μm. (b) Quantitation of *ctgf* mRNA levels in livers of mice expressing YAP (WT), (1SA), (2SA) or (5SA) assayed on the indicated days post-HTVi. Data are the mean ± SD (n=3). (c) Immunoblot by anti-YAP, anti-Myc, anti-P-YAP or anti-GAPDH to detect Myc-tagged YAP in livers of control mice (C) or mice expressing YAP (1SA) or (2SA) assayed on the indicated days post-HTVi. Exo, exogenous; Endo, endogenous. GAPDH, loading control. Uncropped images are shown in Supplementary Fig. 13.

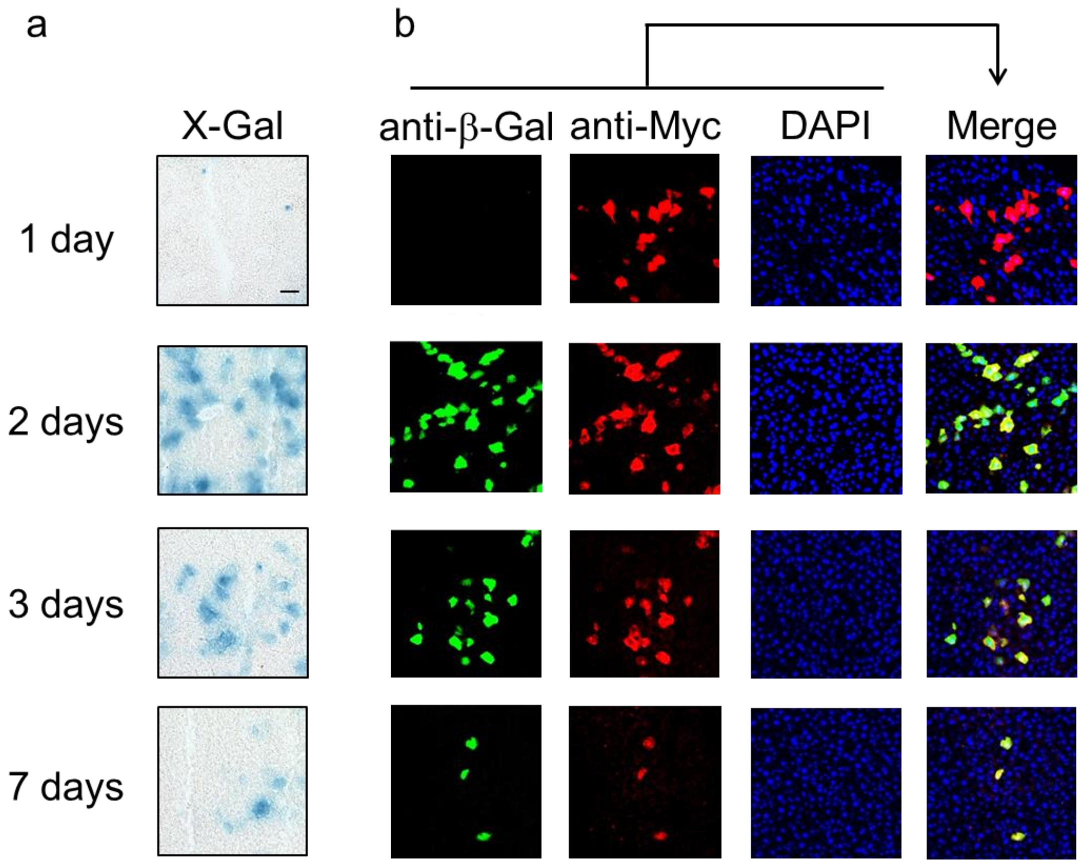

Supplementary Figure 2. Labeling of YAP (5SA)-expressing hepatocytes with β-Gal. (a) Light microscopy to detect X-Gal stained cells in liver sections of ROSA mice expressing Myc-YAP (5SA)-IRES-Cre on the indicated days post-HTVi. Scale bar: 20 μm. (b) Representative confocal immunofluorescence images stained with anti-β-Gal, anti-Myc or DAPI on the indicated days post-HTVi.

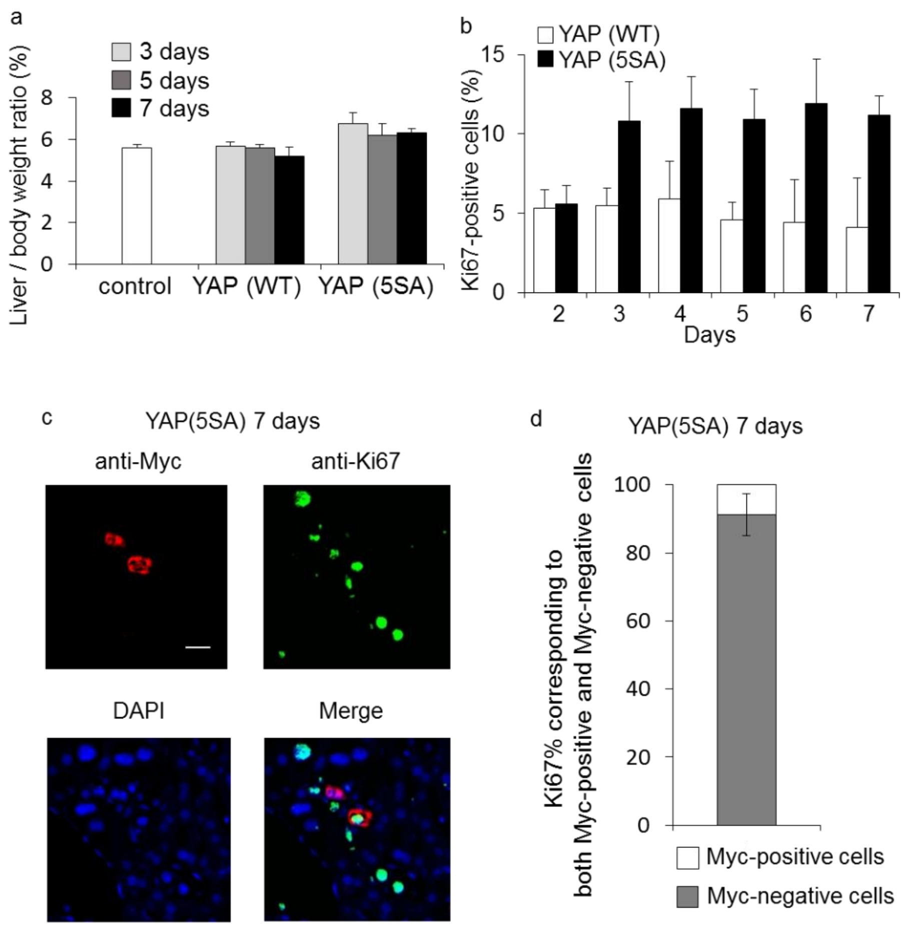

Supplementary Figure 3. Compensatory cell proliferation in YAP (5SA)-expressing liver. (a) Liver-to-body weight ratios in uninjected (Control), YAP (WT)- or YAP (5SA)-expressing mice on the indicated days post-HTVi. Data are the mean ± SD (n=3). (b) Quantification of the percentages of Ki67^{+} cells in the liver sections in (Supplementary Fig. 1d) on the indicated days post-HTVi. Data are the mean ± SD (n=3). (c) Representative confocal immunofluorescence images of cells expressing Myc-tagged YAP as well as proliferating Ki67^{+} cells in YAP (5SA)-expressing livers on day 7 post-HTVi. Nuclei were stained with DAPI. Scale bar: 10 μm. (d) Quantification of the percentages of Myc-negative or Myc-positive cells and Ki67^{+} cells in the liver sections on day 7 post-HTVi. Data are the mean ± SD (n=3).

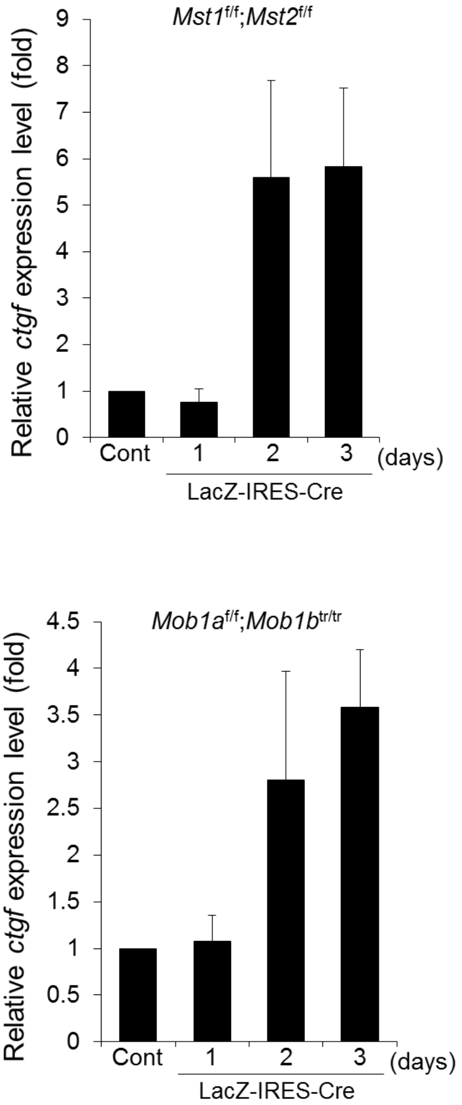

Supplementary Figure 4. Gene expression of *ctgf* in Mst or Mob KO mice.

Quantitation of *ctgf* mRNA levels of liver from *Mst1*$^{f/f}$;*Mst2*$^{f/f}$ or *Mob1a*$^{f/f}$;*Mob1b*$^{tr/tr}$ mice expressing LacZ-IRES-Cre assayed on the indicated days post-HTVi. Data are the mean ± SD (n=3).

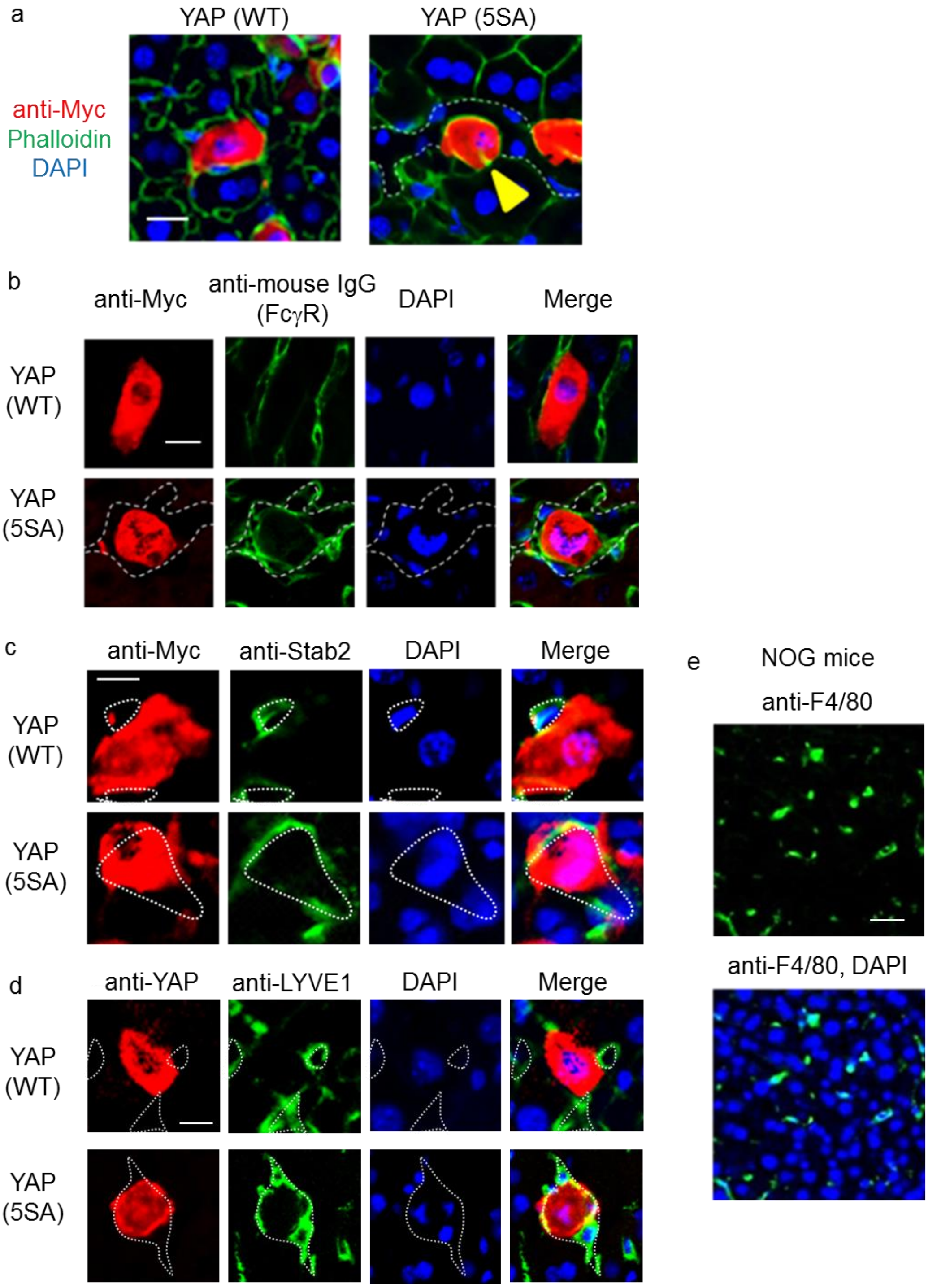
a
YAP (WT)
YAP (5SA)
anti-Myc
Phalloidin
DAPI
b
anti-Myc
anti-mouse IgG (FcγR)
DAPI
Merge
YAP (WT)
YAP (5SA)
c
anti-Myc
anti-Stab2
DAPI
Merge
YAP (WT)
YAP (5SA)
d
anti-YAP
anti-LYVE1
DAPI
Merge
YAP (WT)
YAP (5SA)
e
NOG mice
anti-F4/80
anti-F4/80, DAPI

Supplementary Figure 5. Visualization of sinusoids in YAP (5SA)-expressing liver. (a–d) Representative confocal immunofluorescence images of YAP (5SA)-expressing liver stained to detect Myc-tagged YAP, mouse IgG, Stab2 (a LSEC marker), LYVE1 (another LSEC marker) or DAPI (nuclei). Dashed lines indicate sinusoids. Scale bar: 5 μm. (e) Representative confocal immunofluorescence images of liver sections that were isolated from untreated NOG mice and stained with anti-F4/80 and DAPI. Scale bar: 100 μm.

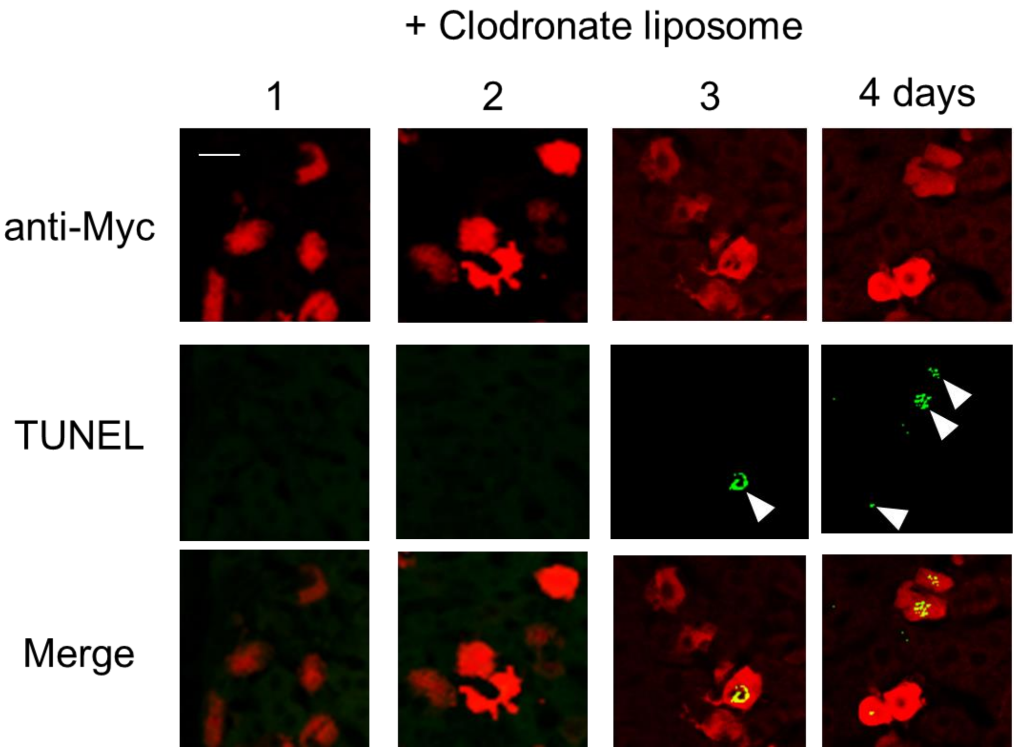

Supplementary Figure 6. Apoptosis of YAP (5SA)-expressing hepatocytes.

Representative confocal immunofluorescence images of livers expressing Myc-tagged YAP (5SA) and treated with clodronate liposomes for the indicated days post-HTVi to deplete Kupffer cells. Sections were stained with anti-Myc or TUNEL. Scale bar: 10 μm.

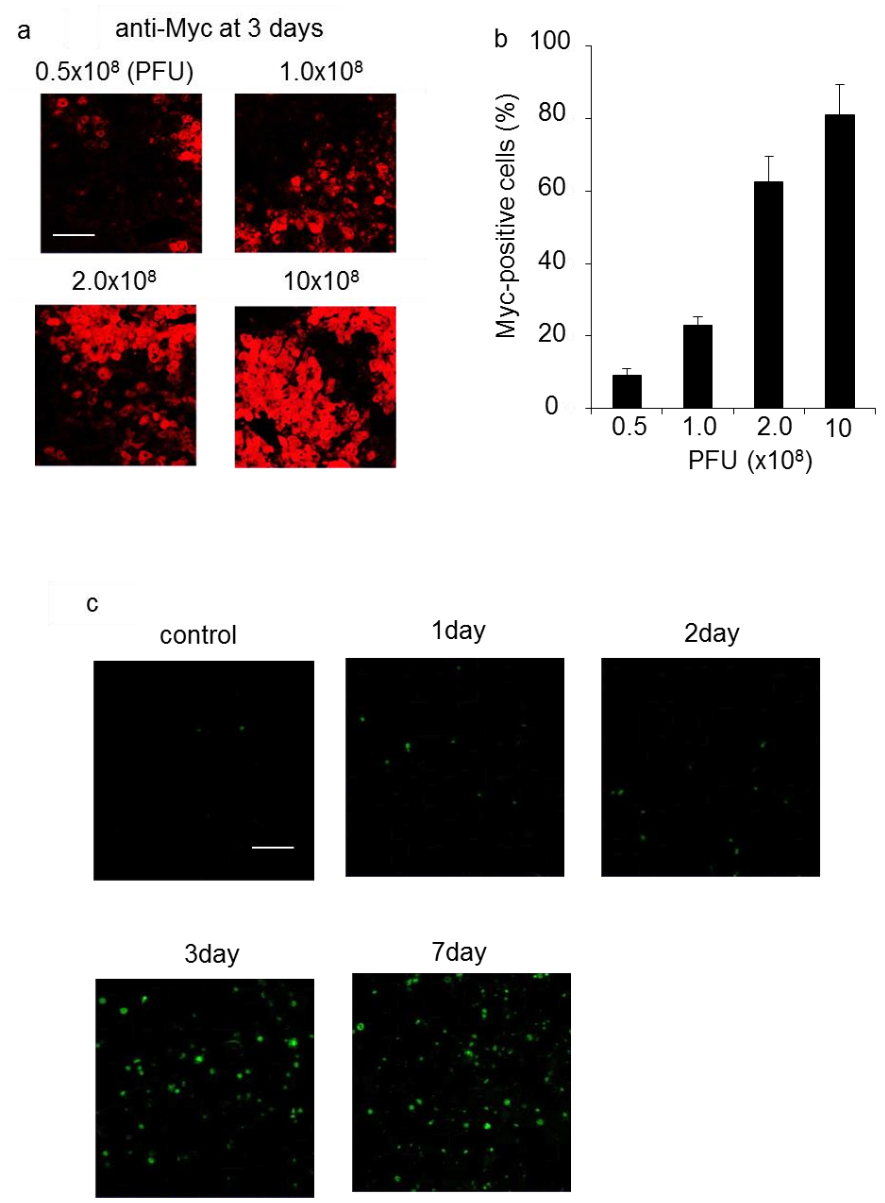

Supplementary Figure 7. The effect of YAP (5SA)-expressing adenovirus infection on hepatocyte proliferation. (a, b) Representative confocal immunofluorescence images (a) and quantification (b) of Myc^+ cells in livers of mice infected with YAP (5SA)-expressing adenovirus vector at the indicated PFU values. Data are the mean ± SD (n=4). (c) Representative confocal immunofluorescence images of $Ki67^+$ cells in liver sections from uninfected (Control) mice and mice infected with YAP (5SA)-expressing adenovirus vector. Livers were assayed on the indicated days

post-infection.

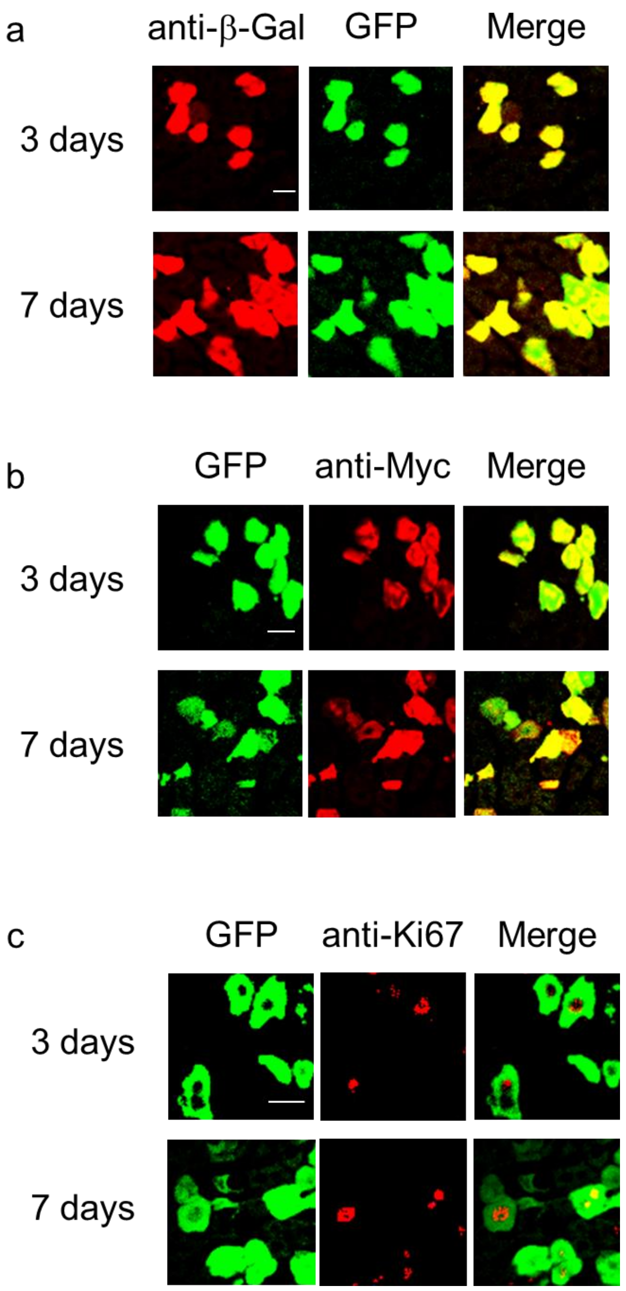

Supplementary Figure 8. Proliferation of YAP (5SA)-expressing hepatocytes. Representative confocal immunofluorescence images of (a) β-Gal$^+$ and GFP$^+$ cells, (b) GFP$^+$ and Myc$^+$ cells and (c) GFP$^+$ and Ki67$^+$ cells in livers of mice infected with YAP (5SA)-expressing and Cre-GFP-expressing adenovirus vectors on the indicated days

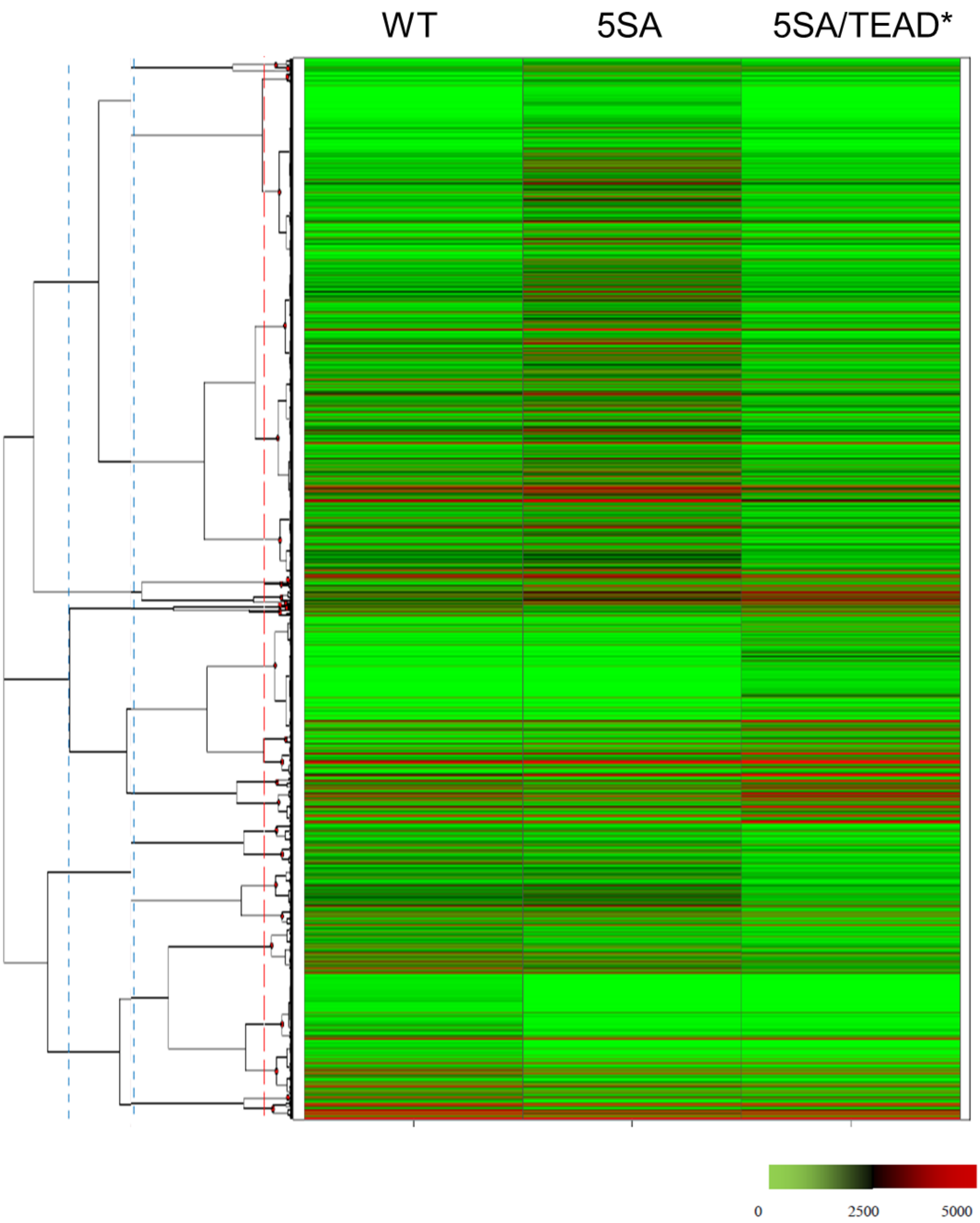

post-infection. Scale bar: 10 μm.

Supplementary Figure 9. Gene expression profiles of YAP (WT)-, YAP (5SA)- or YAP (5SA/TEAD*)-expressing livers. The hierarchical clustering of microarray data for 2,331 microarray probes were obtained from the SurePrint G3 Mouse GE 8x60K Microarray on day 2 after introduction into mice by HTVi and are presented as a

heat-map.

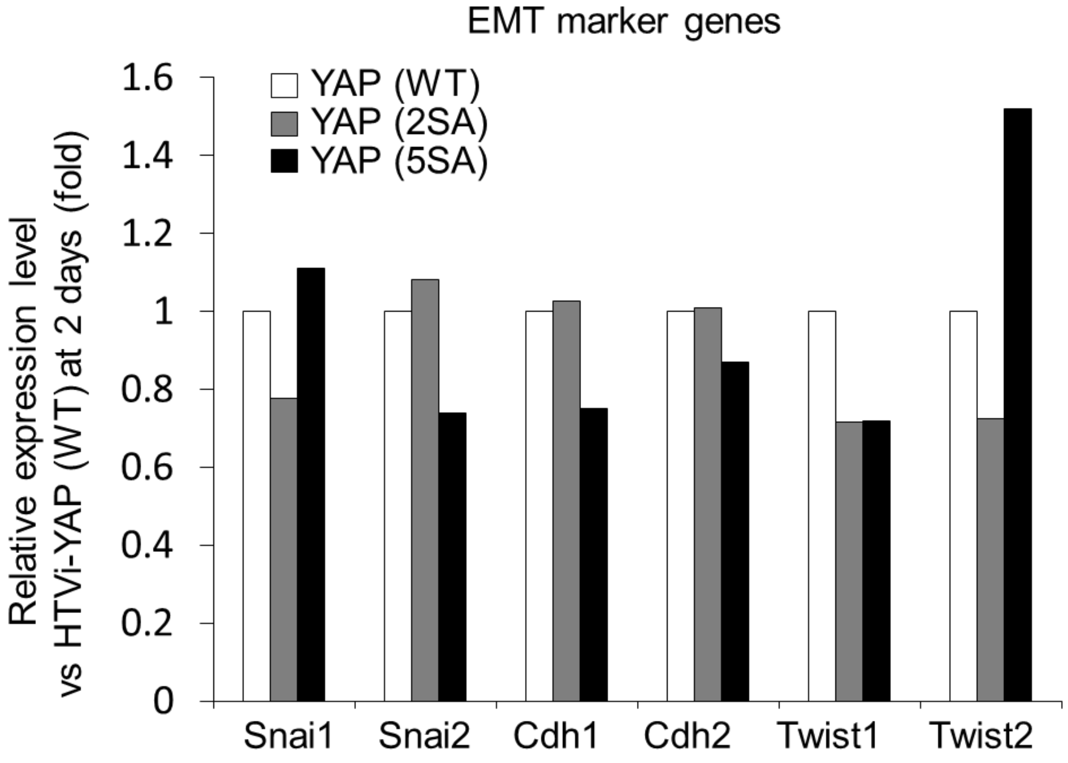

Supplementary Figure 10. EMT marker gene expression as determined by microarray. Quantitation of mRNA levels (as determined by microarray) of the indicated EMT marker genes in YAP (2SA) or YAP (5SA)-expressing livers compared with YAP (WT)-expressing liver on day 2 post-HTVi.

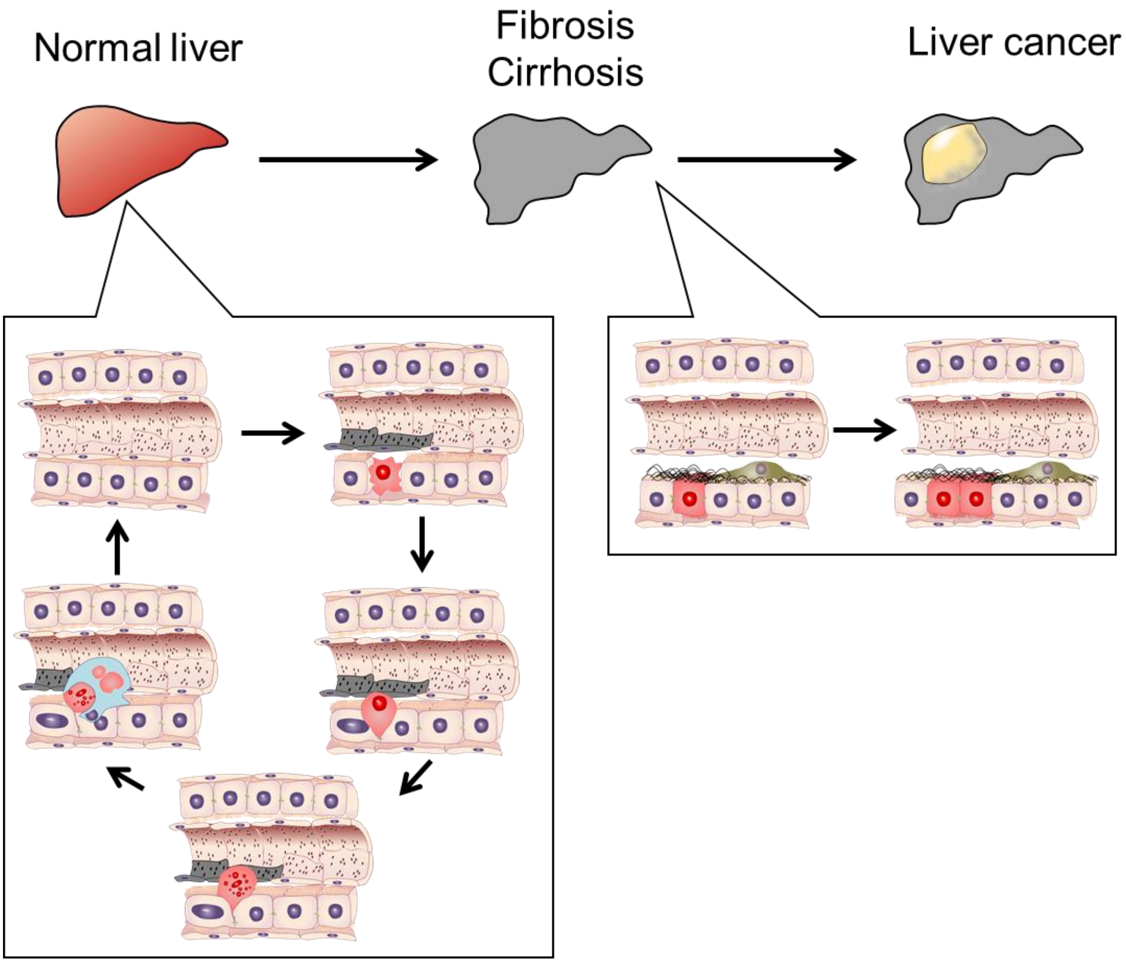

Supplementary Figure 11. Schematic model of responses of hepatocytes expressing activated YAP in normal and diseased livers. YAP-activating hepatocytes proliferate in cases of liver fibrosis/cirrhosis and liver cancer (right panel). On the other hand, hepatocytes in normal liver that sustain damage activate YAP and migrate into the sinusoids, where they undergo apoptosis and are engulfed by Kupffer cells (left panel).

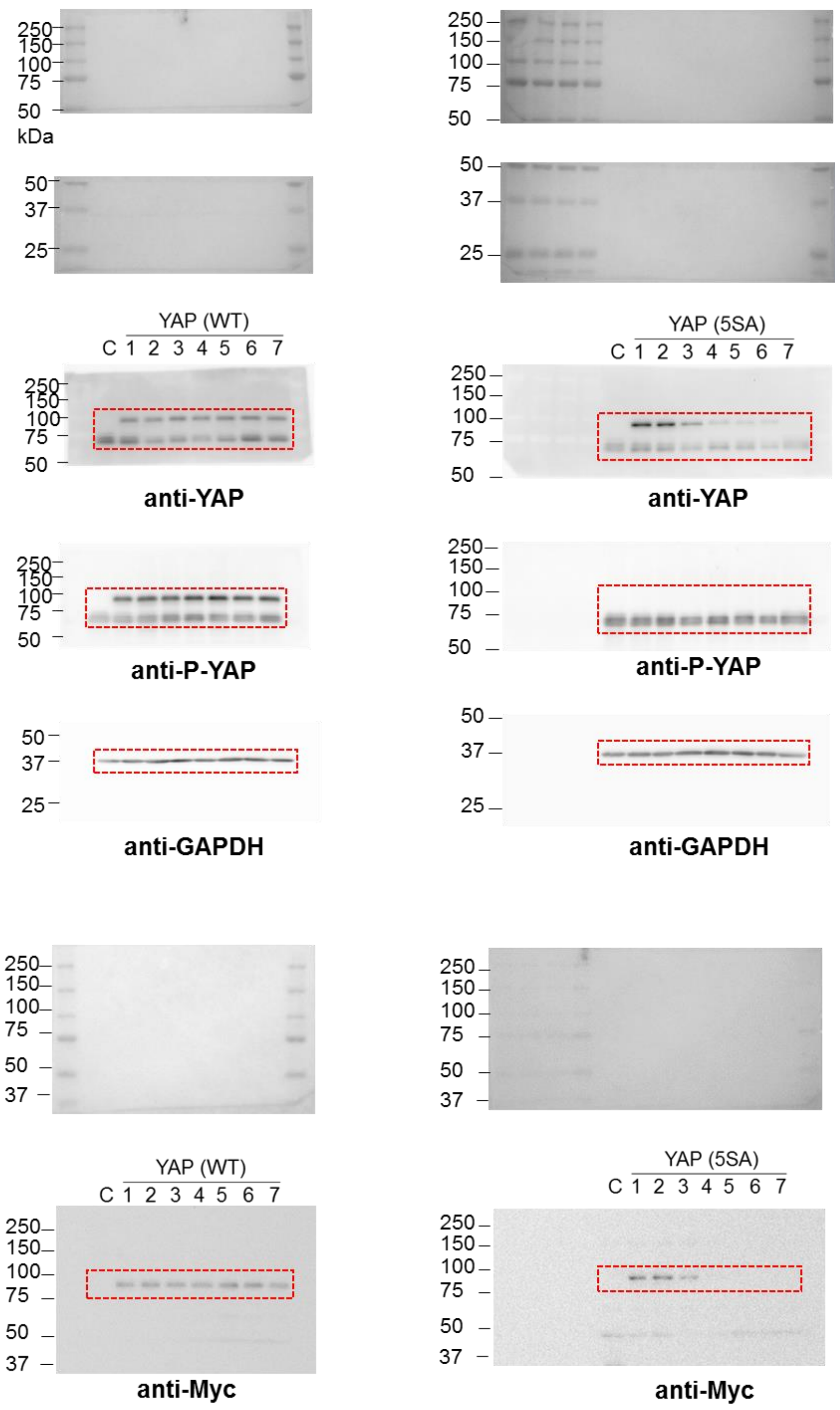

Supplementary Figure 12. Unprocessed scanned images of Figure 1c.

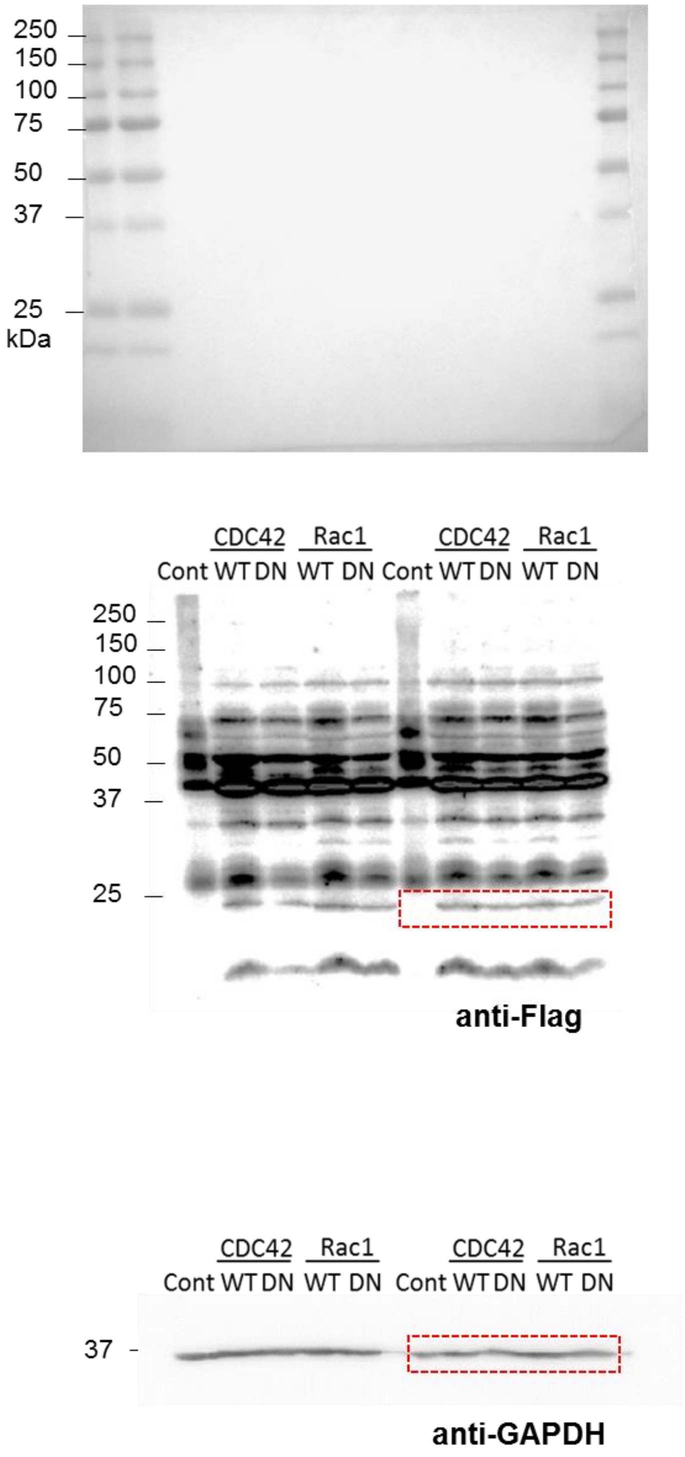

Supplementary Figure 13. Unprocessed scanned images of Figure 4b.

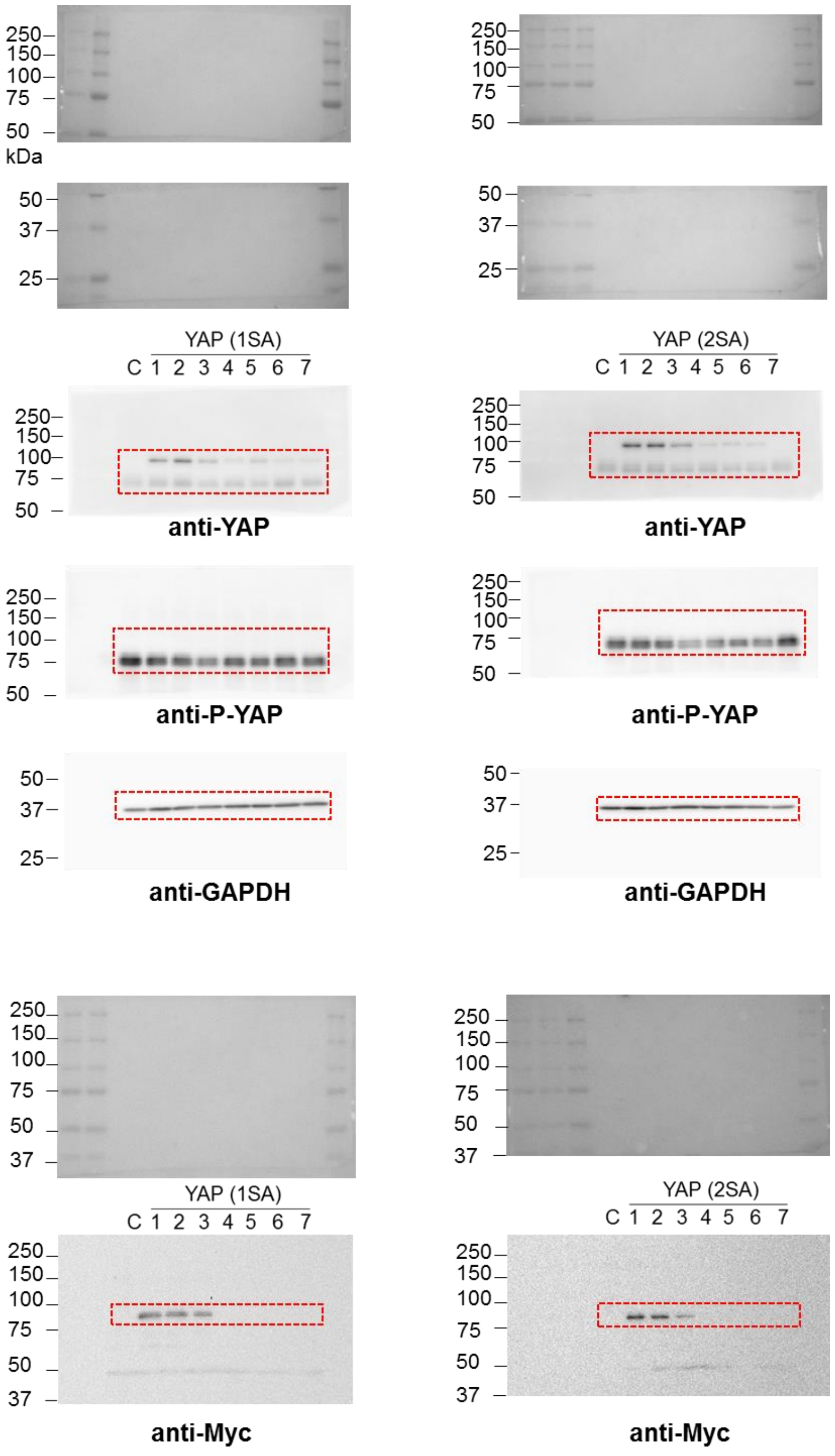

Supplementary Figure 14. Unprocessed scanned images of Supplementary Figure 1c.

Supplementary Table 1.

Gene ontology analysis of genes upregulated by activated YAP.

2 days log2(active-form/WT) > 1.5		
Rank	Gene Ontology	Score(p value)
1	Epithelial cell-cell adhesion	8.05E-04
2	Regulation of Cdc42 GTPase activity (GO:0043088)	1.08E-03
3	Regulation of Cdc42 protein signal transduction (GO:0032489)	1.08E-03
4	Dopamine transport (GO:0015872)	1.23E-03
5	Positive regulation of transmembrane receptor protein serine/threonine kinase signaling pathway (GO:0090100)	1.98E-03
6	Embryonic hindgut morphogenesis (GO:0048619)	3.28E-03
7	Malpighian tubule development (GO:0061327)	3.28E-03
8	Malpighian tubule morphogenesis (GO:0007443)	3.28E-03
9	Regulation of mRNA stability involved in response to stress (GO:0010610)	3.28E-03
10	Catecholamine transport (GO:0051937)	4.05E-03
11	Negative regulation of cell cycle process (GO:0010948)	4.56E-03
12	Cytokine biosynthetic process (GO:0042089)	5.49E-03
13	Cytokine metabolic process (GO:0042107)	5.63E-03
14	Cdc42 protein signal transduction (GO:0032488)	5.87E-03
15	Monoamine transport (GO:0015844)	6.54E-03
